# 

**Published:** 1800

**Authors:** 


### CONTENTS.

Page

I. *A Case of Monstrosity in a Child; with physiological Remarks. By Mr.* W. Simmons, *Member of the Corporation of Surgeons of London; and Senior Surgeon to the Manchester Infirmary.* — — 1

II. *A Description of an improved screw Tourniquet. By the Same.* — — 19

III. *Two Cases of the successful Termination of Wounds that have been hitherto deemed mortal; with Observations. By the Same.* 23

IV. *Case of Retroversion of the Uterus terminating in Abortion and Death. By* James Bell, *M. D. one of the Physicians of the Kelso Dispensary; and formerly President of the Medical and Natural History Societies of Edinburgh.* — — 32

V. *Some Observations relative to the Climate and Diseases of Sierra Leone. By* Thomas Masterman Winterbottom, *M. D. of South Shields, Durham; late Physician to the Settlement at Sierra Leone.* — — 56

VI. *Case of Gangrenous Stomach, with Dysphagia, from Lightning. By Mr.* Patrick Paterson, *Surgeon of the Twenty-ninth Regiment of Light Dragoons.* — — 111

VII. *An*

VII. *An Account of the good Effects of a Decoction of Peach Leaves, in some Affections of the urinary Passages. By Sir* William Bishop, *Knt. Surgeon at Maidstone.* — 122

VIII. *A Case of Lithotomy, attended with some remarkable Circumstances. By Mr.* William Wickham, *Surgeon of the Winchester Hospital.* — — — 126

IX. *Two Cases of Hernia congenita. By Mr.* Henry Fryer, *Surgeon at Stamford.* — 131

X. *Case of imperforate Hymen. By the Same.* 133

XI. *Case of Fungus from a Wound in the Ear. By the Same.* — — 135

XII. *Case of a Wound penetrating the Cavity of the Abdomen. By the Same.* — 137

XIII. *A Case of hairy Concretions found in the human Stomach. By Mr.* William Wood, *Surgeon at Wingham, in Kent; and Fellow of the Linnean Society in London.* — 139

XIV. *A Case of ruptured Uterus, with the Appearances on Dissection. By* Isaac Cathrall, *M. D. Physician at Philadelphia.* 146

XV. *An Account of a ruptured Uterus. By* John Sims, *M. D. Physician in London.* 150

XVI. *Case of Prolapsus Ani; cured partly by an Excision of a Portion of the inner Coat of the Intestine, and partly by the Introduc-*

*tion*

*tion of a Wax Candle within the Cavity of the Rectum.* By Mr. Thomas Whately, *Member of the Corporation of Surgeons of London.* — — — 163

XVII. *Account of the successful Treatment of a large Swelling of the lower Jaw, with an Abscess in the Neck, occasioned by supernumerary Teeth. By the Same.* — — 173

XVIII. *An Account of a Mode of Practice which has been successfully adopted, in Cases of Distortion of the Pelvis, in pregnant Women.* By Mr. John Barlow, *Surgeon at Bolton in Lancashire.* — — 185

XIX. *Observations on the Structure of Hydatids.* By Everard Home, *Esq. F.R.S. From the Philosophical Transactions of the Royal Society of London.* — — 193

XX. *Some Particulars in the Anatomy of the Whale.* By Mr. John Abernethy, *F. R. S. From the same Work.* — — 199

XXI. *Of the Influence of Cold upon the Health of the Inhabitants of London.* By William Heberden, *Jun. M. D. F. R. S. From the same Work.* — — 208

XXII. *Remarks on the Causes and Cure of some Diseases of Infancy.* By Joseph Clarke, *M. D. Licentiate in Physic of the*

*Royal*

*Royal College of Phyſicians in Dublin, and M. R. I. A. From the Tranſactions of the Royal Iriſh Academy.* — — 215

XXIII. *Hiſtory of a Caſe in which very uncommon Worms were diſcharged from the Stomach; with Obſervations thereon. By* Samuel Crumpe, *M. D. M. R. I. A. From the ſame Work.* — — 229

*Index.** — — — 239

* The former Volumes of this Work concluded with a Catalogue of Medical Books; but it is omitted in the preſent; the Publication of this Volume having been unavoidably ſo long delayed that ſuch a Catalogue would have required a much greater Space, than the Editor thought could, with propriety, be allotted to it.

